# Recurrence of Uncomplicated Diverticulitis: A Meta-Analysis

**DOI:** 10.3390/medicina58060758

**Published:** 2022-06-02

**Authors:** Guhyun Kang, Soomin Son, Young-Min Shin, Jung-Soo Pyo

**Affiliations:** 1 Department of Pathology, Daehang Hospital, Seoul 06699, Korea; guhyunkang@daum.net; 2 Division of Molecular Life and Chemical Sciences, College of Natural Sciences, Ewha Woman’s University, Seoul 03760, Korea; smsonaj00@gmail.com; 3 Eulji University School of Medicine, Daejeon 34824, Korea; carlube@naver.com; 4 Department of Pathology, Uijeongbu Eulji Medical Center, Eulji University School of Medicine, Uijeongbu 11759, Korea

**Keywords:** uncomplicated diverticulitis, recurrence, medical treatment, follow-up, meta-analysis

## Abstract

*Background and objective:* This study aimed to investigate the estimated rate and risk of recurrence of uncomplicated diverticulitis (UCD) after the first episode through a meta-analysis. *Methods:* Eligible studies were searched and reviewed; 27 studies were included in this study. Subgroup analyses were performed, based on lesion location, medical treatment, follow-up period, and study location. *Results:* The estimated recurrence rate of UCD was 0.129 (95% confidence interval [CI] 0.102–0.162). The recurrence rates of the right-and left-sided colon were 0.092 (95% CI 27.063–0.133) and 0.153 (95% CI 0.104–0.218), respectively. The recurrence rate according to follow-up period was highest in the subgroup 1–2 years, compared with that of other subgroups. The recurrence rate of the Asian subgroup was significantly lower than that of the non-Asian subgroup (0.092, 95% CI 0.064–0.132 vs. 0.147, 95% CI 0.110–0.192; *p* = 0.043 in the meta-regression test). There were significant correlations between UCD recurrence and older age and higher body temperature. However, UCD recurrence was not significantly correlated with medications, such as antibiotics or anti-inflammatory drugs. *Conclusions:* In this study, detailed information on estimated recurrence rates of UCD was obtained. In addition, older age and higher body temperature may be risk factors for UCD recurrence after the first episode.

## 1. Introduction

Acute diverticulitis develops in 4–25% of patients with diverticulosis [1,2,3]. Uncomplicated diverticulitis (UCD) accounts for 75% of all acute diverticulitis cases [3,4]. Recurrence occurs in approximately one-third of the patients with diverticulitis [2]. Treatment guidelines regarding antibiotics can differ between countries [5,6,7]. Traditionally, treatment of UCD includes bowel rest, intravenous fluids, and antibiotics. However, recent randomized controlled trials (RCTs) have reported the comparison between antibiotics and non-antibiotics therapies in acute diverticulitis [8,9,10]. Avoiding antibiotics as a UCD treatment has been recommended in guidelines based on the results of previous RCTs and other studies [8,11,12,13,14]. However, antibiotics are commonly used in many institutions [15,16]. Estimates from large populations, such as recurrence rates, can be useful for the treatment of UCD in clinical practice. However, conclusive information cannot be obtained from individual studies. Meta-analyses can usefully integrate this information. Although previous meta-analyses have shown odds ratios of recurrence between antibiotics and non-antibiotics [1,17,18], estimated recurrence rates could not be obtained.

The risk of UCD recurrence after treatment remains unclear. The incidence of acute diverticulitis differs between locations and patient age groups [19,20,21]. In addition, recurrence rates may differ according to the patient group. We investigated the recurrence rates of UCD in eligible studies and analyzed the cumulative estimates through a meta-analysis. In the present study, recurrence rates, but not the odds ratio, were estimated as real values. Subgroup analyses were performed for the characteristics of the patients and studies, including lesion location, medication, follow-up period, and study location. In addition, risk of recurrence was evaluated according to various factors.

## 2. Materials and Methods

### 2.1. Published Studies Search and Selection Criteria

**Figure 1:**
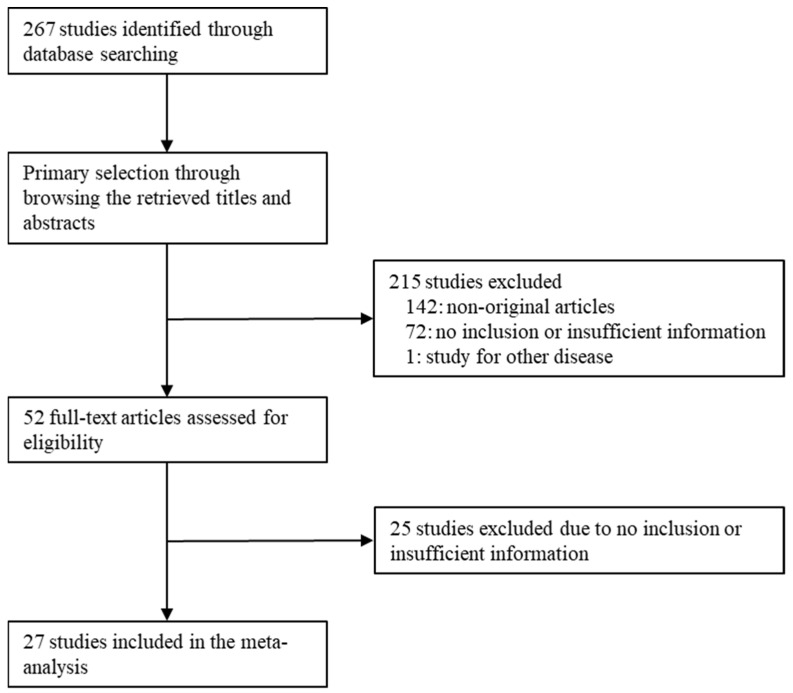
Flow chart of study search and selection methods.

The search for the meta-analysis was performed in the PubMed and the MEDLINE databases through 15 March 2022. The keywords were “uncomplicated diverticulitis” and “recurrence or recur”. Articles with information of the recurrence in UCD were included in the present study. Case reports or non-original articles were excluded. In addition, the articles written in English were included. Detailed characteristics of the 27 eligible studies are shown in Figure 1 and Table 1.

### 2.2. Data Extraction

Two authors independently extracted data from eligible studies. The following data were extracted from all the eligible studies: the family name of the first author, year of publication, study location, number of patients analyzed, study type, lesion locations, medical treatment, and follow-up period [8,9,10,13,22,23,24,25,26,27,28,29,30,31,32,33,34,35,36,37,38,39,40,41,42,43,44]. This study was performed by Preferred Reporting Items for Systematic Reviews and Meta-Analyses (PRISMA).

### 2.3. Statistical Analyses

All data were analyzed using the Comprehensive Meta-Analysis software package (Biostat, Englewood, NJ, USA). The recurrence rates of UCD after the first episode were investigated. Subgroup analyses were performed based on the location of the UCD, treatment, periods of follow-up, study location, and study type. Analysis for heterogeneity between the studies was conducted and evaluated using the Q and I2 statistics and expressed as *p*-values. In addition, the statistical significance of the difference between subgroups was evaluated through the meta-regression test. In the assessment of estimated values, because the eligible studies were evaluated in different populations with variable treatments, the application of a random-effect model rather than a fixed-effect model was more suitable. Publication bias was evaluated using Begg’s funnel plot and Egger’s test. If significant publication bias was found, the degree of publication bias was confirmed through fail-safe N and trim-fill tests. The statistical difference between the subgroups was evaluated through a meta-regression test. The results were considered to be statistically significant at *p* < 0.05.

## 3. Results

### 3.1. Selection and Characteristics of the Studies

A search in the database was performed, and 267 articles were initially found. Through the review of the title and abstract, 52 full-text articles were assessed for eligibility. Finally, 27 articles were included in this meta-analysis. In detail, the causes for the exclusion of the searched articles are shown in Figure 1. Of these, 142 reports were excluded because they were non-original articles. Next, 97 articles were excluded because of insufficient or no information. Another study was excluded due to an article for another disease (*n* = 1). This estimate was obtained from 6731 patients in 27 eligible studies.

### 3.2. The Recurrence Rates of Uncomplicated Diverticulitis

**Figure 2:**
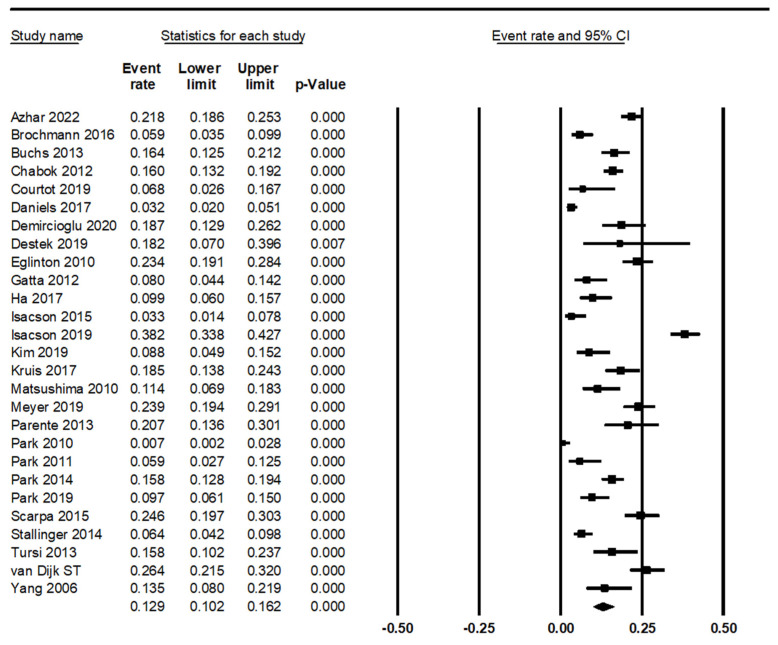
Forest plot for the recurrence rate of uncomplicated diverticulitis.

The estimated recurrence rate of UCD was 0.129 (95% CI 0.102–0.162; Figure 2). Subgroup analyses were performed based on the location of lesions, medical treatment, periods of follow-up, study location, and study type. The recurrence rates in right-and left-sided colons were 0.092 (95% CI 0.063–0.133) and 0.153 (95% CI 0.104–0.218), respectively (Table 2). However, there was no significant difference in the recurrence rates between right- and left-sided colons in the meta-regression test (*p* = 0.062). The recurrence rates of patients with antibiotics or conservative treatments were 0.130 (95% CI 0.096–0.175) and 0.154 (95% CI 0.116–0.202), respectively. The recurrence rate was 0.088 (95% CI 0.045–0.163) in the subgroup with an anti-inflammatory drug. For subgroup analysis based on follow-up periods, subgroups were divided into three categories, such as < 1 year, 1–2 years, and > 2 years. The recurrence rate was higher in follow-up 1–2 years than in other periods. However, there was no significant difference between follow-up periods in the meta-regression test. In subgroup analysis based on study location, the recurrence rates were 0.147 (95% CI 0.110–0.192) and 0.092 (95% CI 0.064–0.132) in Europe and Asia, respectively. There was a statistical significance between the recurrence rates of Europe and Asia subgroups in the meta-regression test (*p* = 0.043).

### 3.3. Comparison of Recurrence of Uncomplicated Diverticulitis According to the Patients’ Characteristics

Next, the risk factors of UCD recurrence were evaluated through comparisons of the odds ratio. Evaluating risk factors included age, sex, white blood cell (WBC) count, C-reactive protein (CRP), body mass index, smoking history, body temperature, multiplicity, and types of medication. Recurrence occurred more frequently in older ages than in younger ages (odds ratio 1.841, 95% CI 1.189–2.851; Table 3). In addition, patients with a high body temperature showed a higher recurrence rate than those with a low body temperature (odds ratio 11.233, 95% CI 1.290–97.824). However, there were no impacts of other risk factors on the recurrence of UCD. Interestingly, patients taking anti-inflammatory drugs showed less frequent recurrence than those not taking anti-inflammatory drugs.

## 4. Discussion

Two RCTs, the AVOD trial and the DIABOLO trial, reported that antibiotics had no significant effect on preventing UCD complications or recurrence [9,10]. However, these RCTs have limitations in interpreting various factors associated with recurrence. In addition, previous meta-analyses did not provide real estimates of recurrence of UCD after treatment. To the best of our knowledge, this is the first meta-analysis to report estimated recurrence rates of UCD after conservative versus medical treatment.

The range of follow-up periods was broad, from four weeks to 11 years in eligible studies. Studies included 22–583 patients. Information from individual studies may vary depending on population or research settings. In Kim’s report, recurrence rates were 9.8% and 7.8% in subgroups with and without antibiotics, respectively [31]. In contrast, Isacson et al. reported a recurrence rate of 31.3% [30]. In theory, a longer follow-up period may be associated with a higher recurrence rate. It can be difficult to draw conclusions from individual studies. This meta-analysis may be useful for obtaining integrated conclusions. In the present study, the estimated recurrence rate was 0.129 (95% CI 0.102–0.162). The estimated recurrence rate ranged from 0.7% to 38.2%. Recurrence can be affected by various factors, such as treatment, location, and follow-up period. Interestingly, the European subgroup had higher recurrence rates than the Asian subgroup (0.147 vs. 0.092; *p* = 0.043 in the meta-regression test). However, there were no significant differences in recurrence rates according to lesion location, medical treatment, or follow-up period.

In the present study, we compared the risk of UCD recurrence according to patient characteristics. Older patients and those with a high body temperature had a significantly higher risk of UCD recurrence than younger patients and those with a low body temperature. In the present study, patients taking anti-inflammatory drugs had an odds ratio of less than 1.000, compared with those not taking anti-inflammatory drugs. However, there was no statistically significant difference between patients treated with and without anti-inflammatory drugs. Other characteristics, such as sex, WBC count, CRP level, smoking history, and multiplicity, had odds ratios higher than 1.000. However, statistical significance was not observed for these factors. In daily practice, evaluation factors obtained from peripheral blood samples can be useful due to the ease of assessment and common laboratory findings. The odds ratio between patients with higher and lower CRP levels was 2.155 (95% CI 0.608–7.643). Although statistical significance was identified in two of the three eligible studies [23,32], the estimated overall odds ratio was not significant. The odds ratios of each study were 6.58 (95% CI 1.05–41.07) and 1.00 (95% CI 1.00–1.01). However, because the CRP levels can be affected by patient conditions and disease progression, more cumulative studies are needed.

Several meta-analyses have reported on UCD recurrence. When recurrence was compared between subgroups with and without antibiotics through meta-analysis [1,17,18], the studies reported no significant difference in the UCD recurrence rates between subgroups. However, the estimated recurrence rate of UCD after antibiotic or conservative treatment could not be obtained from previous meta-analyses. Unlike previous studies, the present study investigated and evaluated estimated recurrence rates. In our results, the overall estimated recurrence rate of UCDs was 0.129 (95% CI 0.102–0.162). In addition, we performed a detailed subgroup analysis based on lesion location, medical treatment, follow-up period, study location, and study type. The odds ratios of the recurrence rates of the medication and non-medication subgroups were similar to those of previous meta-analyses [1,17,18].

This study had several limitations. First, our meta-analysis included various types of studies, such as an RCT, prospective studies, and retrospective studies. Recurrence rates were 0.162 (95% CI 0.094–0.265), 0.110 (95% CI 0.062–0.191), and 0.123 (95% CI 0.093–0.161) in the RCT, prospective study, and retrospective study subgroups, respectively. However, there was no significant difference in recurrence rates between study types in the meta-regression test. A detailed analysis based on various conditions in each study type could not be performed due to insufficient information. Second, eligible studies were mainly conducted in Europe and Asia. Detailed impacts of study location and race on recurrence rates of UCD could not be determined.

## 5. Conclusions

In conclusion, the UCD recurrence rate was estimated to be 12.9%. Recurrence rates were significantly lower in the Asian subgroup, younger patients, and the high body temperature subgroup than in the European subgroup, older patients, and the low body temperature subgroup. In addition, antibiotics treatment has no significant effect on the reduction of recurrence rates in UCD. In this study, we illustrate the recurrence rate that can be expected by each patient’s group.

## Figures and Tables

**Table 1:** Main characteristics of eligible studies.

First Author	Location	Study Type	Follow-Up Period	Lesion	Included Medical Treatment	Number of Patients
Azhar 2022	Sweden	Retrospective	6 mo.	Overall	Antibiotics	583
Brochmann 2016	Norway	Retrospective	12 mo.	Left-side	Antibiotics	220
Buchs 2013	Switzerland	Prospective	24 mo.	Left-side	ND	280
Chabok 2012	Sweden	RCT (AVOD)	12 mo.	Left-side	Antibiotics	582
Courtot 2019	France	Retrospective	33.2 mo.	Right-side	Antibiotics	59
Daniels 2017	Netherlands	RCT (DIABOLO)	6 mo.	Left-side	Antibiotics	528
Demircioglu 2020	Turkey	Retrospective	38 mo.	Overall	Antibiotics	134
Destek 2019	Turkey	Retrospective	2 years	Right-side	Antibiotics	22
Eglinton 2010	New Zealand	Retrospective	101 mo.	Left-side	No	320
Gatta 2012	Italy	Prospective	60 mo.	Overall	Anti-inflammatory	125
Ha 2017	Korea	Retrospective	61 mo.	Right-side	Antibiotics	152
Isacson 2015	Sweden	Prospective	3 mo.	Left-side	No	150
Isacson 2019	Sweden	RCT (AVOD)	11 years	Left-side	Antibiotics	456
Kim 2019	Korea	Prospective	4-6 weeks	Right-side	Antibiotics	125
Kruis 2017	Various	RCT	48 weeks	Left-side	Anti-inflammatory	211
Matsushima 2010	Japan	Retrospective	ND	Overall	Antibiotics	123
Meyer 2019	Switzerland	Retrospective	10 years	Left-side	Antibiotics	301
Parente 2013	Italy	RCT	24 mo.	Left-side	Anti-inflammatory	92
Park 2010	Korea	Retrospective	38 mo.	Right-side	Antibiotics	276
Park 2011	Korea	Retrospective	46 mo.	Right-side	Antibiotics	102
Park 2014	Korea	Retrospective	59 mo.	Right-side	Antibiotics	469
Park 2019	Korea	RCT	ND	Right-side	Antibiotics	176
Scarpa 2015	Switzerland	Prospective	12 mo.	Overall	Antibiotics	256
Stallinger 2014	Italy	Retrospective	3 mo.	Overall	Anti-inflammatory	311
Tursi 2013	Italy	Retrospective	24 mo.	Overall	Antibiotics and Anti-inflammatory	114
van Dijk 2018	Netherlands	RCT (DIABOLO)	24 mo.	Left-side	Antibiotics	468
Yang 2006	Taiwan	Retrospective	37.5 mo.	Right-side	Antibiotics	96

RCT, randomized clinical trial; mo., months; ND, no description.

**Table 2:** The estimated recurrence rate of uncomplicated diverticulitis.

	Number of Subsets	Fixed Effect [95% CI]	Heterogeneity Test (*p*-Value)	Random Effect [95% CI]	Egger’s Test (*p*-Value)	Meta- Regression Test (*p*-Value)
Overall	27	0.190 [0.179, 0.200]	<0.001	0.129 [0.102, 0.162]	<0.001	
Right colon	9	0.120 [0.103, 0.140]	<0.001	0.092 [0.063, 0.133]	0.019	0.062
Left colon	11	0.217 [0.202, 0.232]	<0.001	0.153 [0.104, 0.218]	0.008	
Antibiotics	20	0.196 [0.183, 0.210]	<0.001	0.130 [0.096, 0.175]	0.002	0.741
Anti-inflammatory	5	0.127 [0.101, 0.158]	<0.001	0.088 [0.045, 0.163]	0.122	
Non-antibiotics/ anti-inflammatory	10	0.180 [0.160, 0.202]	<0.001	0.154 [0.116, 0.202]	0.089	
Follow-up < 1 year	9	0.157 [0.143, 0.172]	<0.001	0.102 [0.066, 0.156]	0.010	0.437
Follow-up 1–2 years	5	0.207 [0.180, 0.238]	0.039	0.198 [0.152, 0.252]	0.512	
Follow-up > 2 years	12	0.220 [0.203, 0.238]	<0.001	0.128 [0.086, 0.186]	0.001	
Europe	18	0.207 [0.195, 0.220]	<0.001	0.147 [0.110, 0.192]	0.003	0.043 *
Asia	8	0.120 [0.103, 0.139]	<0.001	0.092 [0.064, 0.132]	0.002	
Randomized clinical trial	7	0.224 [0.206, 0.244]	<0.001	0.162 [0.094, 0.265]	0.080	0.297
Prospective	5	0.166 [0.141, 0.193]	<0.001	0.110 [0.061, 0.191]	0.012	
Retrospective	15	0.171 [0.158, 0.186]	<0.001	0.123 [0.093, 0.161]	0.001	

CI, Confidence interval; * Comparison between the Asian and the non-Asian studies.

**Table 3:** Comparison of odds ratio in the recurrence of diverticulitis according to the patients’ characteristics.

	Number of Subsets	Fixed Effect [95% CI]	Heterogeneity Test (*p*-Value)	Random Effect [95% CI]	Egger’s Test (*p*-Value)
Age (Old vs. Young)	3	1.841 [1.189, 2.851]	0.698	1.841 [1.189, 2.851]	0.417
Sex (Male vs. Female)	7	1.157 [0.925, 1.447]	0.761	1.157 [0.925, 1.447]	0.571
WBC count (High vs. Low)	2	1.010 [0.961, 1.061]	0.907	1.010 [0.961, 1.061]	-
CRP (High vs. Low)	3	2.346 [1.161, 4.741]	0.084	2.155 [0.608, 7.643]	0.868
Body mass index (High vs. Low)	4	0.974 [0.916, 1.035]	0.273	1.016 [0.796, 1.296]	0.731
Smoking history (Yes vs. No)	2	1.487 [0.887, 2.492]	0.913	1.487 [0.887, 2.492]	-
Body temperature * (High vs. Low)	1	11.233 [1.290, 97.824]	1.000	11.233 [1.290, 97.824]	-
Multiplicity (Multiple vs. Single)	3	2.152 [1.355, 3.420]	0.146	1.721 [0.720, 4.115]	0.513
Medication (Yes vs. No)	13	0.950 [0.787, 1.147]	0.797	0.950 [0.787, 1.147]	0.931
Antibiotics	8	1.014 [0.828, 1.241]	0.848	1.014 [0.828, 1.241]	0.063
Anti-inflammatory drug	5	0.639 [0.387, 1.056]	0.795	0.639 [0.387, 1.056]	0.850

CI, Confidence interval; WBC, White blood cell; CRP, C-reactive protein. *, measuring during admission.

## Data Availability

No new data were created or analyzed in this study. Data sharing is not applicable to this article.
